# Towards the Development of Antioxidant Cerium Oxide Nanoparticles for Biomedical Applications: Controlling the Properties by Tuning Synthesis Conditions

**DOI:** 10.3390/nano11020542

**Published:** 2021-02-20

**Authors:** Noemi Gallucci, Giuseppe Vitiello, Rocco Di Girolamo, Paola Imbimbo, Daria Maria Monti, Oreste Tarallo, Alessandro Vergara, Irene Russo Krauss, Luigi Paduano

**Affiliations:** 1 Department of Chemical Sciences, University of Naples Federico II, 80126 Naples, Italy; noemi.gallucci@unina.it (N.G.); rocco.digirolamo@unina.it (R.D.G.); paola.imbimbo@unina.it (P.I.); dariamaria.monti@unina.it (D.M.M.); oreste.tarallo@unina.it (O.T.); alessandro.vergara@unina.it (A.V.); irene.russokrauss@unina.it (I.R.K.); 2 CSGI, Center for Colloid and Surface Science, 50019 Sesto Fiorentino, Italy; 3 Department of Chemical, Materials and Production Engineering, University of Naples Federico II, 80125 Naples, Italy

**Keywords:** cerium oxide, functionalized nanoparticles, wet-chemistry, synthesis condition, redox activity, ROS inhibiting activity

## Abstract

In this work CeO_2_ nanoparticles (CeO_2_-NPs) were synthesized through the thermal decomposition of Ce(NO_3_)_3_·6H_2_O, using as capping agents either octylamine or oleylamine, to evaluate the effect of alkyl chain length, an issue at 150 °C, in the case of octylamine and at 150 and 250 °C, in the case of oleylamine, to evaluate the effect of the temperature on NPs properties. All the nanoparticles were extensively characterized by a multidisciplinary approach, such as wide-angle X-ray diffraction, transmission electron microscopy, dynamic light scattering, UV-Vis, fluorescence, Raman and FTIR spectroscopies. The analysis of the experimental data shows that the capping agent nature and the synthesis temperature affect nanoparticle properties including size, morphology, aggregation and Ce^3+^/Ce^4+^ ratio. Such issues have not been discussed yet, at the best of our knowledge, in the literature. Notably, CeO_2_-NPs synthesized in the presence of oleylamine at 250 °C showed no tendency to aggregation and we made them water-soluble through a further coating with sodium oleate. The obtained nanoparticles show a less tendency to clustering forming stable aggregates (ranging between 14 and 22 nm) of few NPs. These were tested for biocompatibility and ROS inhibiting activity, demonstrating a remarkable antioxidant activity, against oxidative stress.

## 1. Introduction

Among the rare-earth metals, cerium is the most abundant one (it is present at about 66 ppm in the Earth crust) and can exist both in the +3 and +4 oxidation states. Indeed, cerium oxide exists as both CeO_2_ and Ce_2_O_3_ in the bulk state [1,2,3,4]. The stable form of Ce_2_O_3_ is hexagonal, with space group *P3ml*, in which each cerium cation is coordinated with seven oxygen anions. On the other hand, CeO_2_, at room temperature and pressure, crystallizes in the fluorite crystal structure, with space group *Fm3m*. Cerium cations are arranged in a face-centered cubic structure and the O^2−^ anions are positioned in the octahedral interstices. In this structure, each cerium cation is coordinated with eight oxygen anions, while each oxygen anion is coordinated with four cerium cations [4,5].

CeO_2_ is widely used for many technological applications, such as an ultraviolet absorber, catalyst, polishing agent, and gas sensor [6,7,8,9]. In recent years, cerium oxide nanoparticles (CeO_2_-NPs) have attracted great interest in the biomedical field (i.e., as anticancer, antioxidant, and/or antibacterial agent), due to their red-ox properties, which can be opportunely modulated to inhibit (with an antioxidant action) or promote (with a pro-oxidant action) the oxidation processes [3,10,11,12]. More recently, CeO_2_-NPs have acquired an increasing attention due to their peculiar behavior that places them into the promising category of “nanozymes”, which are nanomaterials with enzyme-like features [13,14]. Indeed, they show a superoxide dismutase, catalase and phosphatase mimetic activity, and have been shown the ability to remove Reactive Oxygen Species (ROS) [13,14,15,16].

Generally, CeO_2_-NPs have a crystalline fluorite-like structure, as in the case of bulk material. In the NPs case, crystals usually have some defects due to the coexistence of the trivalent (Ce^3+^) and tetravalent (Ce^4+^) states on the surface [17,18]. In particular, the presence of a different state of oxidation of the cerium oxidation states induces the formation of oxygen vacations that can be easily modulated, either spontaneously or due to the variation of external variables, such as pressure and temperature [18,19,20]. Particularly, CeO_2_-NPs were found to show both antioxidant and pro-oxidant behavior toward different cells, mostly depending on: *i.* the presence of defects (i.e., oxygen vacancies) in the lattice structure, *ii.* the cerium oxidation states (Ce^3+^ or Ce^4+^) on the NPs surface and *iii.* the environmental conditions (such as local pH in cell systems) [14,15,16,21,22,23,24,25]. The presence of defects and the Ce^3+^/Ce^4+^ ratio on the surface are addicted to the NPs shape and size and, therefore, they can be opportunely modulated by varying the synthesis conditions, such as temperature, pressure, nature of the inorganic precursor and/or of the capping agent [13,18]. Particularly, these last components play a key role in the NPs synthesis. The capping agents, also named templating agents, are typically organic molecules that cover the NPs inorganic core. Consequently, the choice of the specific capping agent to employ in NPs synthesis is fundamental in ruling the final size and shape of NPs, and in turn it influences the properties of the inorganic core, both in terms of colloidal stability and functionality [26]. However, the correlation between the alkyl chains length of the capping agent with the final properties of the CeO_2_-NPs, in terms of morphological and optical properties as well as Ce^3+^ formation, is currently not fully clarified.

Generally, NPs presenting a capping agent on the surface show a good solubility in organic solvents. So, an additional step of the surface modification (commonly defined as functionalization) is needed to obtain water-dispersible and biocompatible nanoparticles, which can be used, for example, in the biomedical field [16,27,28]. There are two most common kind of functionalization methods. The first one is based on the addition of an amphiphilic molecule to the external surface of the NPs, without the removal of the capping agent. The hydrophobic interaction between the two amphiphilic molecules creates a two-layer structure exposing a hydrophilic surface, on the surface of the NP, thus allowing the dispersion in water [29,30,31]. The second kind of functionalization is based on the substitution of the capping agent used in the synthesis by replacing it with a bi-functional molecule formed by a functional group able to bind to the NPs surface and a second polar group that makes the compound soluble in water [32,33,34,35].

Understanding the relationship between the synthesis conditions and the physico-chemical properties of the resulting NPs, as well as how these properties can be affected by the surface functionalization, is decisive to make the best use of cerium oxide nanoparticles in any technological field.

In this study, we synthesized CeO_2_-NPs by thermal decomposition of Ce(NO_3_)_3_·6H_2_O salt, varying the reaction temperature and using as capping agents two amines with different alkyl chain, namely octylamine and oleylamine, in order to evaluate the role of different chains lengths in modulating the NP surface properties. The nanoparticles thus obtained were extensively characterized by means of several techniques, such as Wide-angle X-ray Diffraction (XRD), Transmission Electron Microscopy (TEM), Dynamic Light Scattering (DLS), UV-Vis, Fluorescence, Raman and FTIR spectroscopies. The experimental collected data allowed to define the role of the synthesis conditions in affecting the shape and size of nanoparticles as well as optical properties. Furthermore, the CeO_2_-NPs the most promising characteristics (smaller size and better separation of nanoparticles) were functionalized with an amphiphilic molecule, namely sodium oleate (NaOl), in order to disperse them in water. The colloidal stability of functionalized CeO_2_-NPs at different NPs:NaOl ratios was monitored in time with the aim to define the optimal systems with a greater long-term stability in aqueous environment [36,37,38,39,40]. Finally, the selected sample was analyzed through MTT assay on eukaryotic cells in order to evaluate the biocompatibility and the antioxidant activity.

## 2. Materials and Methods

### 2.1. Materials

Cerium(III) nitrate hexahydrate (>99.999% trace metals basis purity), 1-Octadecene (90% technical grade), oleylamine (OL, 70% technical grade), octylamine (OC, 99% purity), chloroform (≥99.5%, contains 100–200 ppm amylenes as stabilizer), sodium oleate (NaOl, 99% purity) were purchased from Sigma Aldrich (Milan, Italy) and used without further purification. All aqueous solutions were prepared using double-distilled Milli-Q water filtered using 0.20 µm filters. Human immortalized keratinocytes (HaCaT) were obtained from Innopront (Biscay, Spain), murine fibroblasts Balb/c-3T3 were from ATCC (Manassas, VA, USA) and were cultured in 10% fetal bovine serum in Dulbecco′s modified Eagle′s medium, in the presence of 1% antibiotics and 2 mM L-glutamine, in a 5% CO_2_ humidified atmosphere at 37 °C.

### 2.2. Synthesis of Alkylamine Coated CeO_2_-NPs

1.74 g of cerium (III) nitrate hexahydrate were dissolved in 25 mL of 1-octadecene at room temperature. In order to have the same precursor salt:capping agent molar ratio equal to 1:3, after the dissolution of the salt, a specific amount of the selected capping agent was added to reactive mixture, as reported in Table 1. Specifically, in order to obtain NPs with different coating, two different capping agents, such as oleylamine (OL) or octylamine (OC), were alternatively used. The obtained solution was placed in a hot bath at 80 °C and stirred for 30 min. Then, the solution was heated to a specific temperature (as reported in Table 1) under an argon atmosphere and stirred for 1 h.

The reaction mixture was then slowly cooled down to room temperature and 30 mL of ethanol were added to induce the precipitation of nanoparticles. The final mixture was transferred to 50 mL centrifuge tubes and centrifuged at 8000 rpm (19712 rcf) for 20 min and the supernatant was separated from CeO_2_-NPs. The NPs were re-dispersed in ethanol and the centrifugation step was repeated at least thrice in order to wash out all the unreacted starting materials. Finally, coated CeO_2_-NPs were dispersed in chloroform.

### 2.3. Functionalization of CeO_2_-NPs with Sodium Oleate

**Figure 1:**
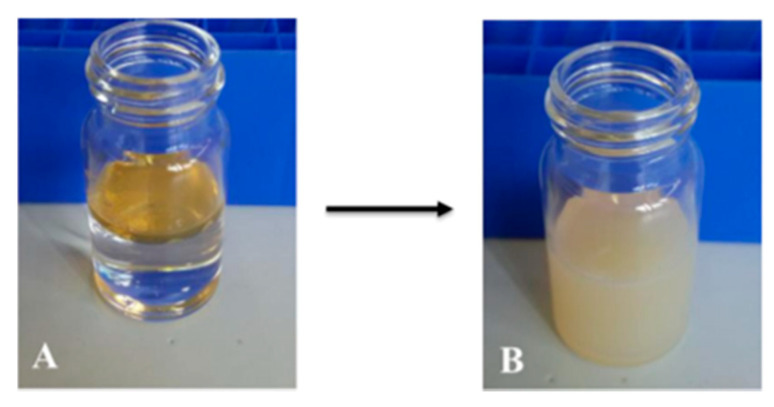
(**A**) Biphasic system formed by aqueous solution of NaOl (bottom) and CeO_2__OL_250 NPs organic solution (top) before sonication and (**B**) final dispersion of functionalized NPs.

The functionalization with sodium oleate, NaOl, was realized only on the nanoparticles coated with oleylamine (OL) and synthesized at 250 °C. First 0.024 g of NaOl were dissolved in 10 mL of bi-distilled water. A certain volume (in the range of 1.25–1.75 mL) of the CeO_2__OL_250 NPs organic solution was added to the aqueous solution of NaOl, determining the formation of a biphasic system, as shown in Figure 1A. Different formulations of coated NPs were prepared by varying the specific volume of the NP organic solution that was put in contact with the amphiphilic molecules, as summarized in Table 2. The biphasic system was sonicated with a tip-sonicator for 5 min to obtain a monophasic system, which was left under stirring overnight, to remove the organic solvent (Figure 1B). In this way amphiphilic coated NaOl/CeO_2_-NPs well dispersed in water were obtained. All the sample preparations are summarized in Table 2.

The determination of the molar ratio NPs:NaOl is based on the determination of the cerium concentration through Inductively Coupled Plasma-Mass Spectrometry (ICP-MS) measurements (see Supplementary Material).

### 2.4. Wide-Angle X-ray Diffraction Analysis (XRD)

Wide-angle X-ray Diffraction (XRD) measurements were carried out to investigate the crystalline structure of CeO_2_ nanoparticles. XRD measurements were performed using nickel-filtered CuKα radiation (λ = 1.5418 Å) with an automatic powder diffractometer (Empyrean by Panalytical, Monza, Italy) operating in the θ/2θ Bragg-Brentano geometry in the range of 10–60°. Approximately, 1 mL of CeO_2_-NPs dispersions was dried to perform XRD analysis.

### 2.5. Dynamic Light Scattering (DLS)

Dynamic Light Scattering (DLS) analysis was employed to determine the size of CeO_2_ nanoparticles as well as to evaluate the possible aggregates formation. DLS measurements were performed using a home-made instrument, composed of a Photocor compact goniometer, an SMD 6000 Laser Quantum 50 mW light source (Quantum Laser, Heaton Mersey, UK) operating at 532.5 nm, a photomultiplier (PMT-120-OP/B), and a correlator (Flex02-01D) from Correlator.com [41,42,43]. All measurements were performed at 25 °C, with the temperature controlled through a thermostatic bath. DLS measurements were performed at fixed scattering angle θ = 120° in the case of CeO_2_-NPs in organic solvent, and at fixed scattering angle θ = 90° in the case of functionalized nanoparticles.

### 2.6. Transmission Electron Microscope (TEM)

Transmission Electron Microscopy (TEM) images were acquired to investigate the morphology of CeO_2_-NPs, using as microscope the FEI TECNAI G2 200 kV (Fei Company, Dawson Creek Drive Hillsboro, Hillsboro, OR, USA). In addition, using ImageJ software, a statistical analysis of the size of the inorganic core was performed. Approximately 10 μL of a given sample was placed on a carbon-coated copper grid and allowed to air dry before imaging.

### 2.7. UV-Visible Spectroscopy

UV-Vis Spectroscopy measurements were performed to determine the absorption properties of coated nanoparticles, by using a Jasco V-560 UV-Vis instrument (JASCO Europe Srl, Lecco, Italy), equipped with a deuterium lamp (190–350 nm) and a halogen lamp (330–900 nm). 1.5 mL of solution containing the coated CeO_2_-NPs was placed in a quartz cuvette in order to carry out the measurements (optical path: 1 cm, band width: 2.0 nm and scanning speed: 40 nm/min).

### 2.8. Fluorescence Spectroscopy

Fluorescence spectra were recorded at 25 °C using a Horiba Scientific Fluoromax-4 spectrofluorometer (Horiba France SAS, JobinYvon, Palaiseau, France) equipped with a Peltier control system and 1 cm path length cells. Each sample was excited to the specific absorption wavelength, determined by means of UV-Vis spectroscopy measurements (integration time: 0.1 s, excitation and emission slit width: 10 nm).

### 2.9. Raman Spectroscopy

Raman spectroscopy has been used as a tool to identify CeO_2_, to provide clues on the degree of nano-structuring [44,45,46,47,48] and to detect the presence of oxygen defects and reduced Ce^3+^ ions [49,50]. Additionally, the organic functionalization to CeO_2_ can be followed via Raman spectroscopy [46]. A confocal Raman microscope (Jasco, NRS-3100, Lecco, Italy) was used to obtain Raman spectra. The 514 nm line of an air-cooled Ar^+^ laser (Melles Griot, 35 LAP 431–220, Carlsbad, CA, USA) was injected into an integrated Olympus microscope and focused to a spot size of approximately 2 µm by using a 100× objective, with a final 2 mW laser power at the sample. A holographic notch filter was used to reject the excitation laser line. Raman scattering was collected by using a Peltier-cooled 1024 × 128 pixel CCD photon detector (Andor DY401BVI, Andor technology oxford instruments, Belfast, UK). For most systems, it took 60 s to collect a complete data set. Measurements were at least triplicated for scope of reproducibility.

### 2.10. FTIR Spectroscopy

The functionalization of CeO_2_ was followed by means of Fourier-transform infrared (FTIR) spectroscopy [48]. Infrared spectra were collected with an FTIR Nicolet 5700 (Thermo Fisher Scientific, Waltham, MA, USA), in the range of 4000–600 cm^−1^, using the Omnic software. The spectrometer is equipped with a KBr beam splitter and a MCT-B detector. Spectra were recorded in ATR mode with a ZnSe crystal by accumulating 64 scans, with a resolution of 4 cm^−1^.

### 2.11. Cell Culture and MTT Assay

Cells were seeded in 96-well plates at a density of 2.5 × 10^3^/well. 24 h after seeding, increasing concentrations of NaOl/CeO_2_-NPs (0.65–3.25 nM) were added to the cells for 48 h. At the end of the incubation, cell viability was assessed by the tetrazolium salt colorimetric assay (MTT), as described by Sobel et al. [51]. Cell survival was expressed as the percentage of viable cells in the presence of CeO_2_-NPs compared to control cells, represented by the average obtained between untreated cells and cells supplemented with the highest concentration of NaOl. Each sample was tested in three independent analyses, each carried out in triplicates.

### 2.12. DCFDA Assay

The antioxidant effect of nanoparticles, against oxidative stress, was determined by measuring the intracellular Reactive Oxygen Species (ROS) levels as reported by Petruk et al. [52]. Briefly, HaCaT cells were pretreated for 2 h with either NaOl/CeO_2_-NPs, oleylamine or sodium oleate. Then oxidative stress was induced by incubating cells with 300 µM sodium arsenite (SA) for 1 h at 37 °C. Fluorescence intensity was measured by a Perkin-Elmer LS50 spectrofluorometer (Perkin-Elmer, Waltham, MA, USA) (525 nm emission wavelength, 488 nm excitation wavelength, 300 nm/min scanning speed, 5 slit width for both excitation and emission). ROS production was expressed as the percentage of DCF fluorescence intensity of the sample under test, with respect to the untreated sample. Three independent experiments were carried out, each one with three determinations.

All the results are presented as the mean of results obtained after three independent experiments (mean ± SD) and compared by one-way ANOVA according to the Bonferroni′s method (post hoc) using Graphpad Prism for Windows, version 6.01.

## 3. Results

Cerium oxide nanoparticles (CeO_2_-NPs) were successfully synthesized through thermal decomposition of cerium (III) nitrate hexahydrate (Ce (NO_3_)_3_·6H_2_O) at 150 °C, using either octylamine (OC) or oleylamine (OL) as capping agent. Moreover, in order to study the effect of temperature, the characteristics of CeO_2_-NPs synthesized with oleylamine at either 150 °C or 250 °C were compared. Finally, the promising NPs, i.e., those with the less poly-dispersion and the smallest size, were functionalized with sodium oleate (NaOl). In the following sections we present the details of the characterization of the NPs at each stage of the process from synthesis to the obtainment of water dispersible nanoparticles.

### 3.1. NP Characterization

**Figure 2:**
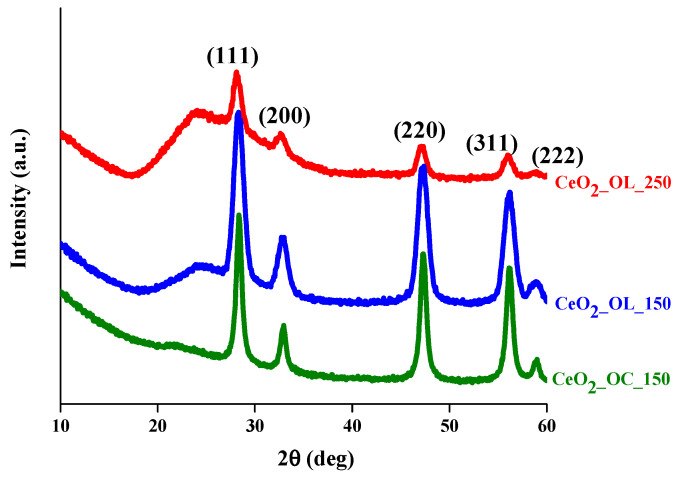
XRD patterns of CeO_2__OC_150 (green), CeO_2__OL_150 (blue) and CeO_2__OL_250 (red).

Characterization of the inorganic core of CeO_2_ NPs was carried out by means of Wide-angle X-ray Diffraction (XRD). The XRD patterns of CeO_2_-NPs prepared with octylamine at 150 °C (CeO_2__OC_150), and with oleylamine at 150 °C (CeO_2__OL_150) or at 250 °C (CeO_2__OL_250) are shown in Figure 2.

The values of the diffraction angles (2θ) and Miller indices (hkl) of the peaks observed in the profiles of Figure 2 are listed in Table 3. These patterns show the typical peaks of a face centered cubic structure (JCPDS 36-1451) [53,54,55]. The broad halo at 2θ values of less than 28° could be due to the presence of the organic coating on the surface of CeO_2_-NPs.

**Figure 3:**
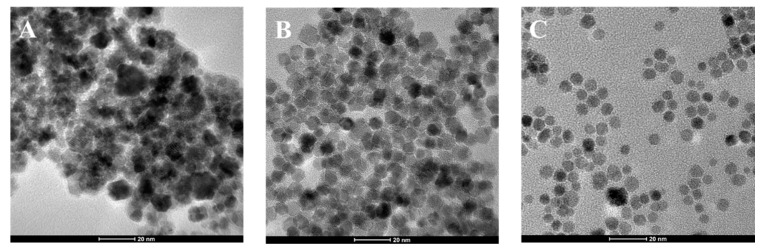
(**A**) TEM image of CeO_2__OC_150, (**B**) TEM image of CeO_2__OL_150 and (**C**) TEM image of CeO_2__OL_250 NPs (scale bar: 20 nm).

Morphological features of all three NP systems were analyzed by means of TEM (Figure 3). In particular, representative TEM images indicate that CeO_2__OC_150 nanoparticles present an irregular shape (Figure 3A), CeO_2__OL_150 NPs are characterized by a hexagonal shape (Figure 3B), while CeO_2__OL_250 ones have a spherical shape (Figure 3C). Moreover, while in the latter system NPs appear well separated, TEM images of CeO_2__OC_150 and CeO_2__OL_150 highlight their tendency to self-assemble with formation of large clusters.

Statistical analysis of TEM images allowed us to determine the mean radius of the NP inorganic core and the relative standard deviation, which is about 6.5 ± 0.1 for CeO_2__OC_150, 3.5 ± 0.1 nm for system CeO_2__OL_150 and 2.5 ± 0.1 for sample CeO_2__OL_250.

**Figure 4:**
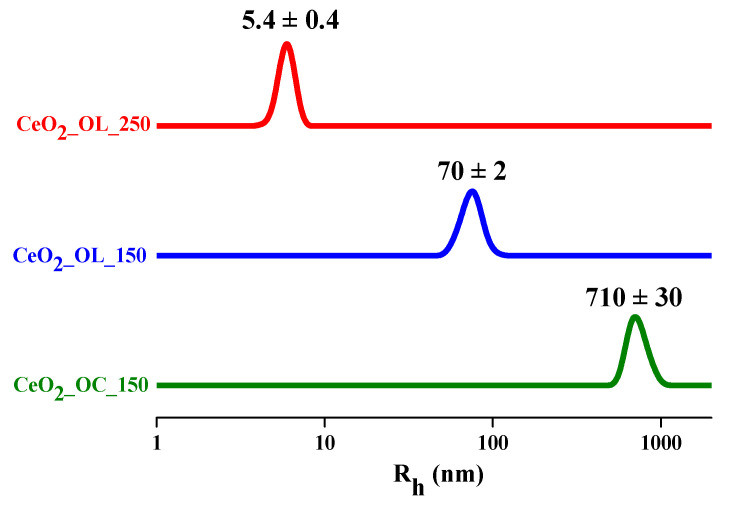
Hydrodynamic radius distribution of CeO_2__OC_150 (green), CeO_2__OL_150 (blue) and CeO_2__OL_250 (red).

In order to investigate the dimension of NPs as a whole, i.e., including the organic capping agent layer, the NP hydrodynamic radius R_h_ by means of Dynamic Light Scattering (DLS) was determined. In all cases DLS profiles reported in Figure 4 present a single distribution centered at about 710 nm for CeO_2__OC_150 (green line) at about 70 nm for CeO_2__OL_150 (blue line) and at about 5 nm for CeO_2__OL_250 (red line). The R_h_ values determined for CeO_2__OC_150 and CeO_2__OL_150 confirm the presence of clusters observed by TEM images, and of single nanoparticles for CeO_2__OL_250. Furthermore, taking into account the R_h_ value and the core dimension as determined by TEM, thickness of the coating layer of about 2.5 nm was calculated, in agreement with oleylamine length in its full-length extension (2.0 nm) [56].

### 3.2. Coating Characterization

To unambiguously identify CeO_2_ core and to detect the presence of oxygen defects and reduced Ce^3+^ ions, Raman spectroscopy was employed. This analysis also provided clues on the nano-structuring degree of coated NPs and on the main features of the organic layer, which was better investigated, also thanks to evidence provided by FTIR spectroscopy.

**Figure 5:**
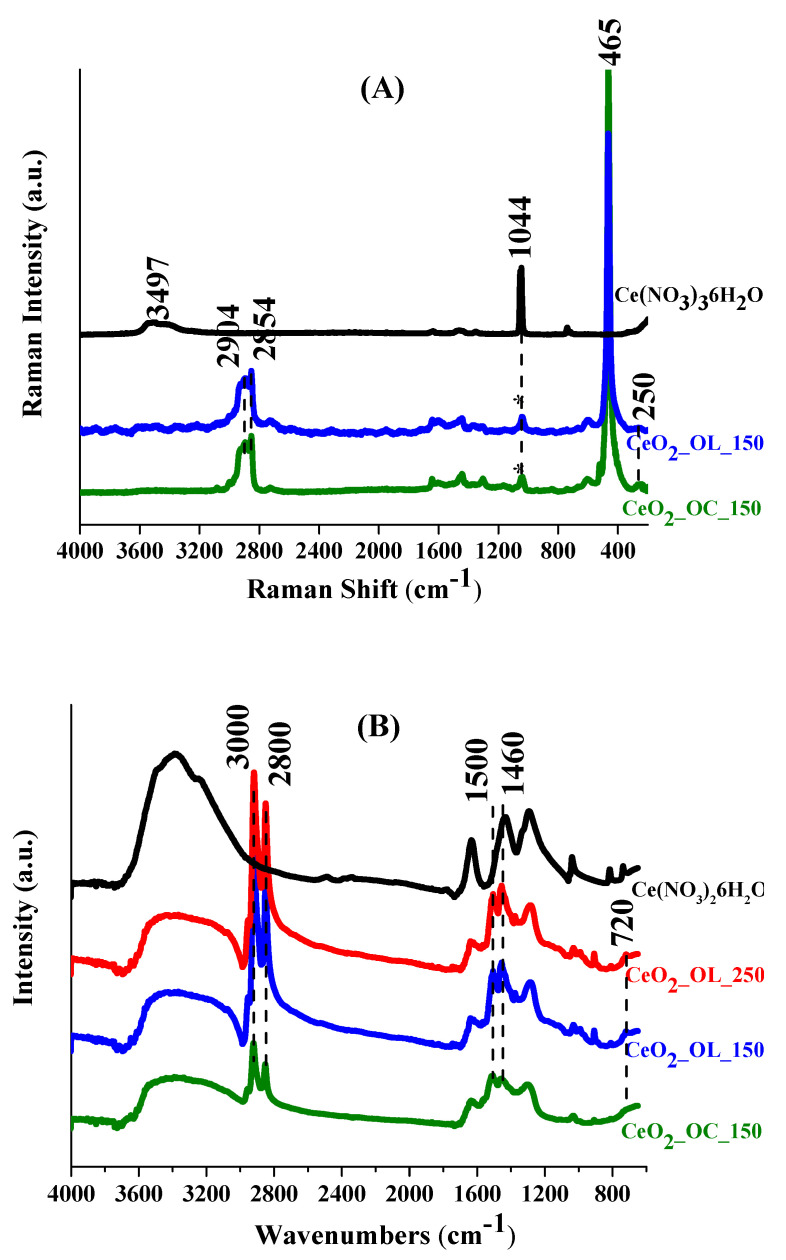
Vibrational characterization of CeO_2_ nanoparticles. (**A**) Raman spectra of CeO_2__OC_150 (green line), CeO_2__OL_150 (blue line) and inorganic precursor (black line). (**B**) IR spectra of CeO_2__OC_150 (green line), CeO_2__OL_150 (blue line), CeO_2__OL_250 (red line), and inorganic precursor (black line).

Raman spectra were recorded for all NP systems, as well as on precursor Ce(NO_3_)_3_ ·6H_2_O for comparison (Figure 5A). Ce(NO_3_)_3_·6H_2_O is characterized by a band at 1044 cm^−1^ (starred in Figure 5A), clearly visible in the spectra of NPs too, indicating a residual content of the precursor in NP samples. On the other hand, the major band around 465 cm^−1^ in all the NP spectra indicates formation of CeO_2_. In particular, this Raman band can be attributed to the symmetrical stretching mode of CeO_8_ cubes (F_2g_ mode) [46]. F_2g_ mode for CeO_2__OC_150 and CeO_2__OL_150 samples shows very similar Raman shifts (at 464 and 465 cm^−1^, respectively), indicating a close similarity of nano-structuring.

Additional minor Raman features could appear in CeO_2_ when significant O vacancies and reduction of Ce^4+^ to Ce^3+^ occurred. Indeed, such a local symmetry distortion generates the additional 550 and 598 cm^−1^ bands [48]. In our case the analysis of the 598 cm^−1^ peak, possibly related to Ce^3+^ presence, is hindered by the overlapping signals due to OL and OC components (data not shown). A 250 cm^−1^ band is observed in both CeO_2__OC_150 and CeO_2__OL_150 samples, and it is particularly pronounced in the former one (Figure 5A). This band is elsewhere assigned to a surface mode of the clean CeO_2_(111) surface [49]. Due to a high fluorescence, only the CeO_2_ major band can be clearly detected in CeO_2__OL_250 Raman spectra and compared to that of the other systems (see Figure S1 in Supplementary Information). The wavenumber of the F_2g_ band of CeO_2__OL_150 and CeO_2__OL_250 are 465 and 461 cm^−1^. It is reported that the highest change in the F_2g_ wavenumber occurs at low nanoparticle size (a 8 cm^−1^ increase in Raman shift going from 5 to 8 nm), followed by a flat trend of the Raman shift at nanoparticle size higher than 9 nm [48]. Therefore, the observed variation from 465 to 460 cm^−1^ suggests some reduction of the nanoparticle size increasing the temperature from 150 to 250 °C, in agreement with the results of TEM analysis that revealed a reduction from 7 to 5 nm (Figure 3). The inset on the CeO_2_ F_2g_ mode in Figure S1 shows also how its increase in wavenumber is accompanied by a corresponding decrease in bandwidth, consistently with previous observations on F_2g_ mode CeO_2_ [48].

Unfortunately, it was possible to investigate only a limited infrared spectral region ranging between 600 and 4000 cm^−1^ (Figure 5B), due to instrumental limitations. This prevents from seeing to observe the major band at 450 cm^−1^ corresponding to cerium oxide [48]. However, a band at 720 cm^−1^ (Figure 5B) is observed, that can be related to another band corresponding to cerium oxide reported at 770 cm^−1^ [57]. The observed 50 cm^−1^ shift could be due to the presence of the organic coating.

Intense bands in the 2800–3000 cm^−1^ region, due to CH stretching, and 1460–1500 cm^−1^ region, due to CH_2_ bending in both Raman and FTIR spectra (Figure 5A,B) confirm a significant presence of the organic coating. Since Raman spectra show bands from both CeO_2_ and OL/OC, a quantitative comparison between the two functionalization protocols can be achieved: the ratio between the intensity of the major CH stretching envelope (2800–3000 cm^−1^) and the CeO_2_ major band at 465 cm^−1^ is definitively higher for the OC than for the OL sample. This allows inferring that the presence of OC in the synthesis leads to increased coverage of the nanoparticle surface.

### 3.3. Spectroscopic Properties

**Figure 6:**
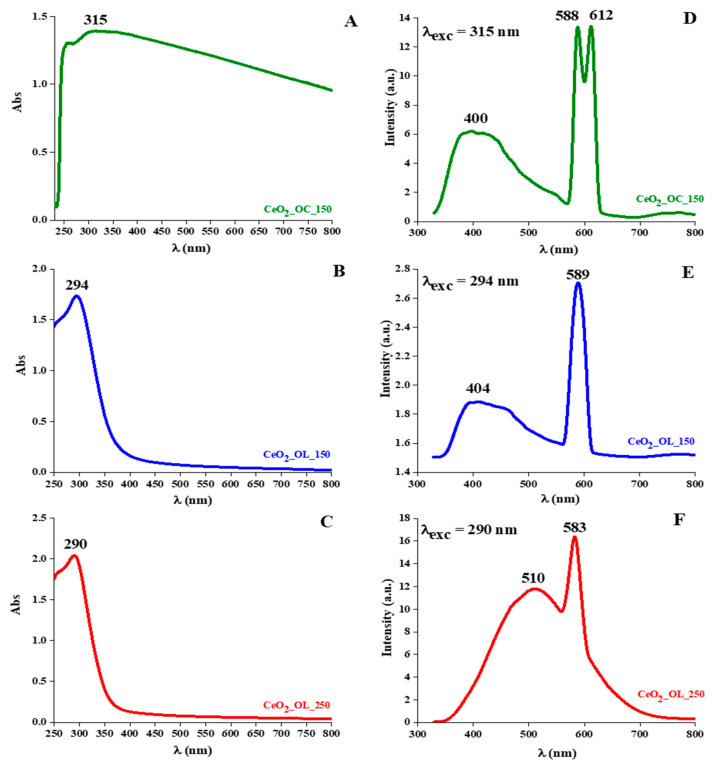
(**A**) UV-Visible spectra of CeO_2__OC_150 (green line), (**B**) CeO_2__OL_150 (blue line) and (**C**) CeO_2__OL_250 (red line); (**D**) Fluorescence spectra of CeO_2__OC_150 (green line), (**E**) CeO_2__OL_150 (blue line) and (**F**) CeO_2__OL_250 (red line).

Spectroscopic properties of CeO_2_ NPs were investigated by means of UV-Vis and fluorescence spectroscopy. As shown in Figure 6, the UV-Vis spectrum of CeO_2__OC_150 sample is characterized by a wide absorption that extends also to the visible region (A). On the other hand, the spectra of CeO_2_ NPs with oleylamine show a well-defined peak in the UV region, with a maximum centered at λ = 294 nm in the case of CeO_2__OL_150 (B) and at 290 nm in the case of CeO_2__OL_250 (C).

Fluorescence spectra were recorded in the 350–800 nm range by exciting each sample at the maximum absorption wavelength as determined by UV-Vis measurements, that are 315, 294 and 290 nm for CeO_2__OC_150, CeO_2__OL_150 and CeO_2__OL_250, respectively (Figure 6). CeO_2__OC_150 nanoparticles present a broad band centered at 400 nm and an unusual double peak in the yellow-orange region with maxima at 588 and 612 nm (panel D). CeO_2__OL_150 system presents the same broad band centered at 404 nm and an intense single peak in the yellow region centered at ~589 nm (panel E). Finally, for CeO_2__OL_250 samples, the peak in the yellow region is just slightly blue-shifted with respect to CeO_2__OL_150, being positioned at 583 nm, while the broad band at smaller wavelengths is significantly red shifted being centered at 510 nm (panel F).

### 3.4. Functionalization of CeO_2_-NPs with Sodium Oleate

**Figure 7:**
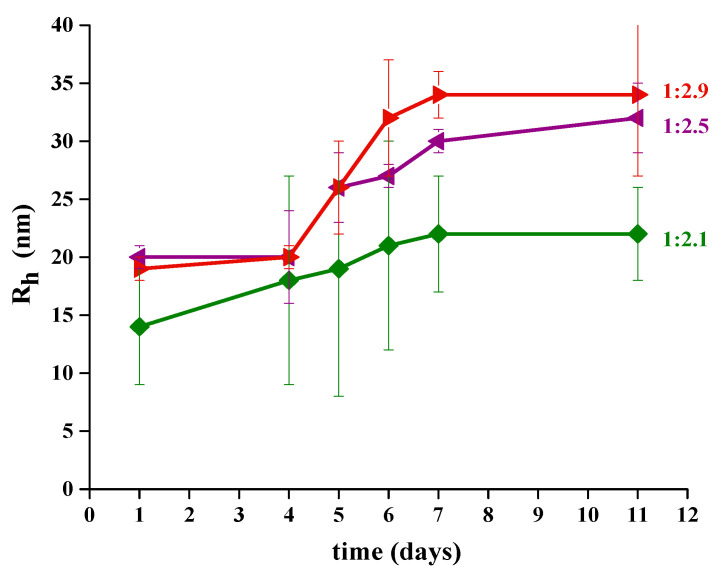
Hydrodynamic radius distribution over time of CeO_2__OL_250 NPs with different molar ratio NPs:(NaOl × 10^6^).

CeO_2_-NPs synthesized at 250 °C using oleylamine (OL) appear as monodispersed single spherical NPs, not forming clusters. Consequently, they were chosen to be functionalized with sodium oleate, NaOl. This step was carried out with the aim to confer a colloidal stability to the synthesized CeO_2_-NPs in an aqueous environment. Different weight ratios between NaOl and CeO_2_-NPs were considered, as summarized in Table 2, with the aim to identify the best conditions to obtain a stable system. The time evolution of functionalized NaOl/CeO_2_-NPs in aqueous solution was followed by means of DLS and was monitored for several days. The time evolution of R_h_ for all samples is reported in Figure 7. Even after few days from the preparation all the samples are quite stable and present a single main population, whose R_h_ ranges between 14 and 20 nm (shown Figure S2). As time goes by a moderate increase of the hydrodynamic radius is observed. Notably, the sample at molar ratio NPs:NaOl 1:(2.1 × 10^6^) shows aggregates with smaller size and with less increase with time.

### 3.5. Evaluation of Biocompatibility of NaOl/CeO_2_-NPs on Eukaryotic Cells

**Figure 8:**
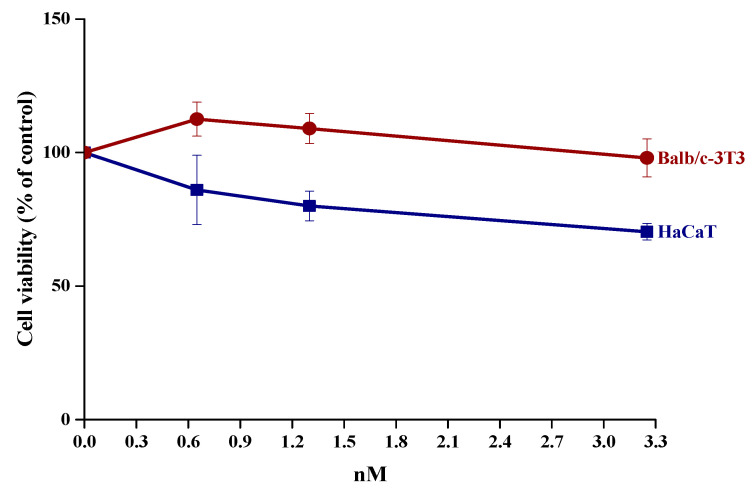
Effect of CeO_2_-NPs on the viability of HaCaT (wine circles) and Balb/c-3T3 (blue squares) cells after 48 h incubation with increasing concentration of nanoparticles (0.65–3.25 nM). Cell viability was assessed by the MTT assay, and cell survival expressed as percentage of viable cells in the presence of nanoparticles under test, with respect to control cells grown in the absence of nanoparticles. Data shown are means ± S.D. of three independent experiments.

To verify the not-toxicity of the functionalized CeO_2_-NPs nanoparticles, as fundamental requirement for a perspective use in the biomedical field, biological tests were carried out on a selected sample in the presence of eukaryotic cells. The sample selected for this purpose had a NPs:NaOl molar ratio of 1:(2.1 × 10^6^), because of a small number of NPs in the final cluster. The biocompatibility of water soluble NaOl/CeO_2_-NPs was analyzed by a dose-response test on immortalized human keratinocytes (HaCaT) and murine fibroblasts (Balb/c-3T3). Cell viability was assessed by the MTT assay, and cell survival was expressed as the percentage of viable cells in the presence of NPs compared to that of control samples. As show in Figure 8, no cytotoxic effect was observed when cells were incubated with NPs under all the analyzed conditions.

### 3.6. Determination of Intracellular ROS Levels

**Figure 9:**
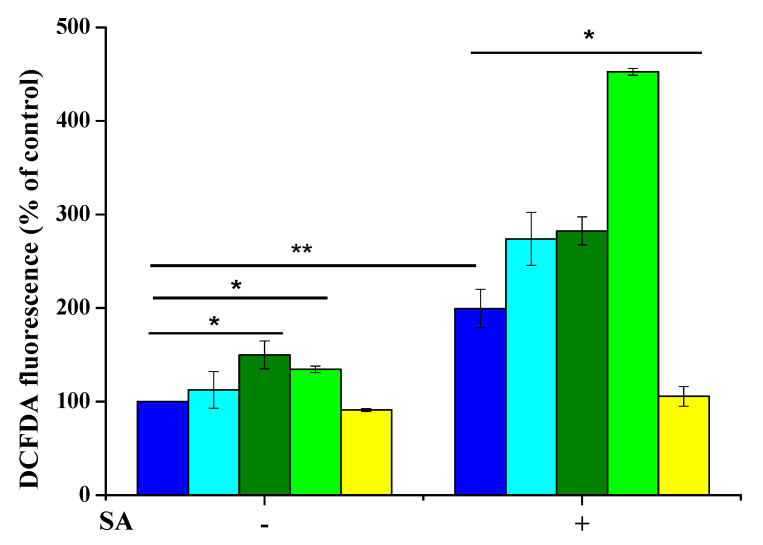
Determination of intracellular ROS levels by DCFDA assay on HaCaT cells. Cells were preincubated in the presence of 1.3 nM CeO_2__OL_250 (yellow bars), 7.3 µM oleylamine (light blue), 7.1 µM sodium oleate (dark green bars), and a mixture of oleylamine and sodium oleate (light green bars) for 2 h, prior the induction of oxidative stress. Blue bars are referred to untreated cells in the absence (−) or in the presence (+) of 300 µM sodium arsenite (SA). Values are expressed as percentage with respect to control (i.e., untreated) cells. Data shown are means ± S.D. of three independent experiment. * indicates *p* < 0.05, ** indicates *p* < 0.005.

The antioxidant activity of functionalized CeO_2_-NPs was evaluated on a cell-based system. HaCaT cells were incubated with 1.3 nM of NaOl/CeO_2_-NPs. In parallel experiments, cells were treated with either 7.3 µM oleylamine or 7.1 µM sodium oleate or a mixture of both compounds, in order to verify if the capping agent or the functionalizing agent could affect the antioxidant activity of the nanoparticles. These concentrations correspond to those present on the NP surface. After 2 h incubation, oxidative stress was induced by treating cells with 300 µM sodium arsenite (SA) for 1 h. Indeed, SA is known to induce oxidative stress by increasing ROS production, oxidative DNA damage and finally apoptosis [52]. Here, ROS levels were measured by using H_2_DCF-DA as a probe. For each set of experiments, untreated cells were used as a control. As shown in Figure 9, no effect on ROS levels was observed when cells were incubated with NaOl/CeO_2_-NPs for 2 h (yellow bars) in the absence of oxidative stress, whereas a significant alteration in ROS production was observed in the presence of sodium oleate (dark green bars) and the mixture of oleylamine and NaOl (light green bars). SA treatment significantly increased DCF fluorescence intensity in untreated cells (blue bars), as well as in cells preincubated with OL (light blue bar, on the right), NaOl (dark green bar, on the right) or the mixture of the two (light green bar, on the right). Noteworthy, when cells were pretreated in the presence of NPs prior to SA exposure, increase in ROS production was observed (yellow bar, on the right).

## 4. Discussion

In this work, the synthesis and physico-chemical properties of cerium oxide nanoparticles (CeO_2_-NPs), obtained by thermal decomposition of Ce(NO_3_)_3_·6H_2_O salt using as capping agent either octylamine or oleylamine, were proposed with the aim to mainly define the role of the alkyl chain length affecting the NP properties. In addition, CeO_2_-NPs in the presence of oleylamine were also prepared at either 150 °C (as in the case of octylamine) or 250 °C to evaluate the effect of the synthesis temperature. Structural features of all the systems are summarized in Table 4.

For all samples, Raman spectra reveal the formation of cerium oxide, as confirmed by the band at 465 cm^−1^, although a residual presence of the precursor used in the synthesis was observed at least for systems obtained at 150 °C. At the same time XRD measurements (Figure 2) confirm that CeO_2_-NPs have the typical fluorite structure. Moreover, the very similar position of Raman band for NPs obtained with either octylamine or oleylamine at 150 °C indicates that they have similar size, in agreement with the NPs radii determined by TEM (Table 4). Moreover, comparing the dimensions of the systems obtained by DLS (Table 4), it can be deduced that the presence of oleylamine allows a better dispersion of the NPs with respect to the systems composed by bare nanoparticles and, in all cases, aggregates of smaller dimensions are obtained. Indeed, the appropriate choice of the molecule to be used as capping agent is decisive to control and modulate the size as well as the aggregation properties of CeO_2_ nanoparticles. For example, Keller et al. obtained clusters of about 230 nm by self-aggregation of bare CeO_2_ [37]. Similarly, Römer et al. obtained aggregates of bare CeO_2_-NPs with dimensions ranging between 170 and 350 nm which reduced to few nm only in the presence of a PVP coating [58]. On the other hand, the use of different capping agents leads to the formation of larger aggregates, as observed by Oriekhova et al. [38], which covered the CeO_2_-NPs with fulvic acids obtaining systems of about 100 nm. However, our results demonstrate that the alkyl chain length of the capping agent as well as the synthesis temperature do not affect only the NP size but also its shape. In particular, TEM images (Figure 3) indicate that a longer capping agent determines the formation of NPs with a well-defined hexagonal morphology, differently from the irregular shapes obtained in the case of the shorter octylamine. Considering the temperature effect, keeping fixed the capping agent nature, we observe that an increase of temperature from 150 to 250 °C induces a morphological change from hexagonal to spherical shape and, most importantly, hinders the formation of aggregates. Therefore, the use of an amine with a longer chain allows us to better control the NP shape and size, while an increase of the synthesis temperature can be employed to obtain a better separation of the NPs. Interestingly, although both Raman and FTIR analyses confirm the presence of organic coating for all three systems, a greater coating efficiency is found in the case of octylamine with respect of oleylamine, indicating that the crucial factor is not the grade of coverage but the length of the capping agent. It is important to mention that oleylamine impurities may induce significant luminescence in Raman investigation of NPs (particularly using blue laser lines [59]. In our case, oleylamine treated samples showed weak luminescence for the sample at 150 °C (Figure 5A) and a significant one for 250 °C sample. Nevertheless, in our study the 514 nm line excitation produced Raman spectra for oleylamine-treated samples (both at 150 and 250 °C) good enough to compare fine features of the major CeO_2_ Raman band (wavenumber and bandwidth) in the different preparations (Figure S1).

Optical properties are strongly influenced by the use of different capping agents and temperatures: the use of octylamine, in the place of oleylamine, causes strong absorption even in the visible region; this last evidence has been interpreted as a major presence of Ce^3+^ with respect to Ce^4+^ species [53,54,55,56,60]. This evidence can also be the cause of a peak splitting in the fluorescence spectrum. The presence of this peak splitting could be due to Rabi splitting phenomena, which were until now observed for metallic NPs (i.e., Au or Ag) and, only partially, for some semiconductors [60,61,62,63,64,65,66,67]. In our case, the splitting was observed only for CeO_2__OC_150 samples and could be related to the presence of oxygen vacancies on the nanoparticles surface (intraparticle voids) and/or to the formation of cluster (interparticle voids), that are indeed observed by means of both TEM and DLS. In addition, a broad band in the 400–500 nm region appears in fluorescence spectra of all three systems. This band, whose position varies slightly with the capping agent but significantly with the synthesis temperature, can be attributed to the presence of defects and to the hopping from different defect levels as well [68,69].

CeO_2_-NPs can effectively be made water-soluble with a further functionalization process based on hydrophobic interactions between the alkyl tails of the oleylamine as capping agent and the amphiphilic sodium oleate, confirming a successful strategy to functionalize nanoparticles [40,70,71]. The idea to use sodium oleate represents a not-common choice to decorate CeO_2_-NPs prepared by thermal decomposition method, thus proposing as a promising approach to obtain stable formulations. Indeed, this functionalization induces the formation of NP aggregates with dimensions ranging between 14 and 35 nm, depending on the NPs:NaOl molar ratio, which show a good colloidal stability up 11 days. Therefore, the functionalization with organic molecules increases the stability of the system in the time, going from few hours, as also observed by Keller et al. [36] and Tso et al. [35], to many days in the present work. In addition, the functionalization protocol developed in this work allows obtaining aggregates of NPs of a smaller size than those obtained in other protocols proposed [40,70,71].

Finally, the functionalized CeO_2_-NPs prepared with the optimal NPs:NaOl ratio 1:(2.1 × 10^6^) show a good stability over time and the formation of small clusters (Figures S2 and S3 in Supplementary Materials). On this basis, such a sample represented the best formulation of NaOl/CeO_2_-NPs to be tested for biomedical applications. Indeed, as demonstrated by MTT assay, these show a high biocompatibility on two immortalized cell lines, such as HaCaT and Balb/c-3T3, exerting a significant antioxidant activity counteracting the SA-induced stress by inhibiting the ROS production.

## 5. Conclusions

The present study sheds light on the role of the nature of the capping agent, in terms of length of the alkyl tail, and the synthesis temperature in tuning the formation of CeO_2_-NPs with a desired size or morphology. The synthesis carried out at 250 °C and in the presence of oleylamine produces regular NPs of about 5 nm of radius characterized by a low clustering tendency, with a well-defined peak in the UV region, due to a major amount of Ce^4+^ with respect to Ce^3+^, differently from what observed for CeO_2_-NPs synthesized at 150 °C and in the presence of octylamine. These results suggest that the use of oleylamine as capping agent promotes the Ce^4+^ formation. Such nanoparticles were made water soluble by employing a functionalization protocol based on hydrophobic interaction and use of NaOl, obtaining rather stable NP aggregates of quite small size, particularly when the NPs are synthetized with long tail oleylamine (about 4–5 single NPs) which promote a more stable organic bilayer on the nanoparticles surface. Functionalized nanoparticles result biocompatible and able to inhibit ROS production in cells where oxidative stress was induced, opening the way to their development for applications in biomedical field. Overall, we obtained water soluble and quite stable small aggregates of CeO_2_-NPs, whose properties and aggregation we aim at regulating in the future by fine-tuning functionalization parameters. Thus, they can be well considered promising for further development in different fields, including the biomedical one.

## Figures and Tables

**Table 1:** Experimental parameters for coated CeO_2_-NPs synthesis. Variation of capping agent and/or temperature results in different size and shape of NPs without changing the core nature.

	OC_150	OL_150	OL_250
Temperature (°C)	150	150	250
Capping agent (mL)	1.98	3.95	3.95

**Table 2:** Summary of the different samples of cerium oxide nanoparticles synthesized with oleylamine at 250 °C (CeO_2__OL_250 NPs) and functionalized with a constant amount of sodium oleate (NaOl).

Molar Ratio NPs:(NaOl × 10^−6^)	CeO_2__OL_250 NPs in Chloroform (mL)	Sodium Oleate (g)	Water (mL)
1:2.1	1.25	0.024	10
1:2.5	1.50	0.024	10
1:2.9	1.75	0.024	10

**Table 3:** Experimental values of the diffraction angles (2θ, degs) and corresponding Miller indices (hkl) of diffraction peaks observed in the profiles reported in Figure 2.

	(111)	(200)	(220)	(311)	(222)
CeO_2__OC_150	28.31°	32.98°	47.20°	56.18°	58.92°
CeO_2__OL_150	28.31°	32.83°	47.20°	56.18°	58.92°
CeO_2__OL_250	28.16°	32.69°	47.20°	56.03°	58.92°

**Table 4:** CeO_2_ NP structural and spectroscopic features.

	Crystalline Structure	Shape	<R> (TEM) (nm)	Rh (DLS) (nm)	Absorption Maximum (nm)	Emission Maximum (nm)
CeO_2__OC_150	Fluorite	Irregular	6.5 ± 0.1	Cluster 710 ± 30	315	400, 588, 612
CeO_2__OL_150	Fluorite	Hexagonal	3.5 ± 0.1	Cluster 70 ± 20	294	404, 589
CeO_2__OL_250	Fluorite	Spherical	2.5 ± 0.1	5.4 ± 0.4	290	510, 583

## Data Availability

The data in this study is available on reasonable request from the corresponding author.

## Supplementary Materials

The following are available online at https://www.mdpi.com/2079-4991/11/2/542/s1, Figure S1: Raman spectra for the three CeO_2_ samples: Green CeO_2__OC_150 (ν = 464 cm^−1^; Δν = 20 cm^−1^), Blue CeO_2__OL_150 (ν = 465 cm^−1^; Δν = 20 cm^−1^), Red CeO_2__OL_250, (ν = 461 cm^−1^; Δν = 23 cm^−1^); Figure S2: Hydrodynamic radius distribution of CeO_2__OL_250 NPs functionalized with a constant amount of sodium oleate at first day after preparation; Figure S3: Hydrodynamic radius distribution over time of sample NPs:NaOl molar ratio 1:(2.1 × 10^6^).
