# Supplementary material for: Towards the Development of Antioxidant Cerium Oxide Nanoparticles for Biomedical Applications: Controlling the Properties by Tuning Synthesis Conditions
Source: Nanomaterials (Basel). 2021 Feb 20;11(2):542. doi: 10.3390/nano11020542 (PMC7924622; doi:10.3390/nano11020542)
Supplement: Supplementary file 1 [file nanomaterials-11-00542-s001.pdf]

Supplementary Information

Towards the development of antioxidant cerium oxide nanoparticles for biomedical applications: controlling the properties by tuning synthesis conditions.

Noemi Gallucci,[1,2] Giuseppe Vitiello,[2,3,*] Rocco Di Girolamo,[1] Paola Imbimbo,[1] Daria Maria Monti,[1] Oreste Tarallo,[1] Alessandro Vergara,[1] Irene Russo Krauss,[1,2] Luigi Paduano [1,2,*]

[1] Department of Chemical Sciences, University of Naples Federico II, 80126 Naples, Italy
[2] CSGI, Center for Colloid and Surface Science, 50019 Sesto Fiorentino (FI), Italy
[3] Department of Chemical, Materials and Production Engineering, University of Naples Federico II, 80125 Naples, Italy
* Giuseppe Vitiello, Email: giuseppe.vitiello@unina.it
Luigi Paduano, Email: lpaduano@unina.it

Evaluation of NP concentration

Ce concentration in nanoparticles samples was determined by ICP-MS in terms of mg/kg concentration. Considering a CeO_2 density of 7.6 g/cm^3 and the volume of NP core as experimentally determined by TEM images it was possible to estimate the weight of a single nanoparticle being 10^{-19} g, corresponding to a nanoparticle molecular weight of about 299545.6 g/mol. We thus converted Ce concentration into NP molar concentration and used this value to determine the oleylamine and sodium oleate concentration needed for coating [1-2].

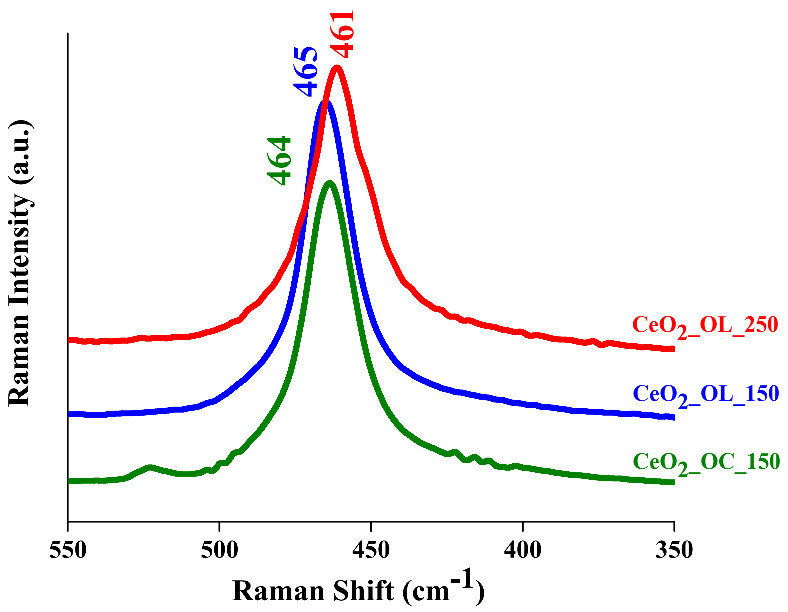

Figure S1. Raman spectra for the three CeO_2 samples: Green CeO_2_OC_150 (ν=464 cm^{-1}; $\Delta\nu$=20 cm^{-1}), Blue CeO_2_OL_150 (ν=465 cm^{-1}; $\Delta\nu$=20 cm^{-1}) , Red CeO_2_OL_250, (ν=461 cm^{-1}; $\Delta\nu$=23 cm^{-1}).

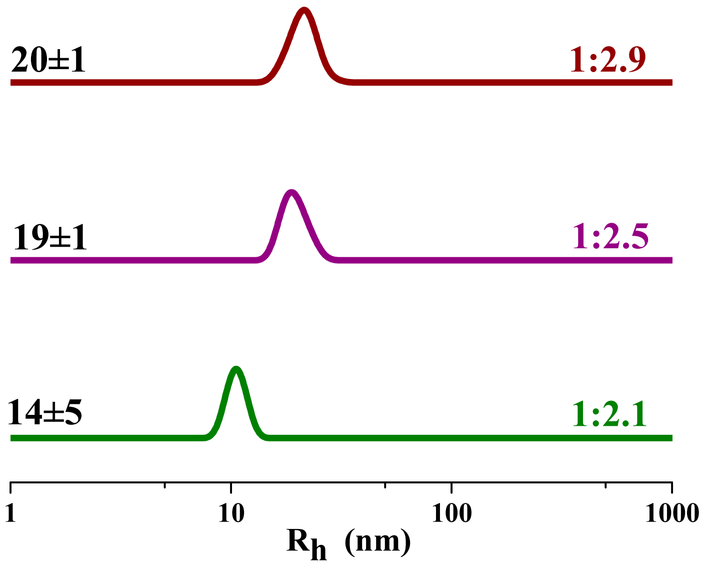

Figure S2. Hydrodynamic radius distribution of CeO_2_OL_250 NPs functionalized with a constant amount of sodium oleate at first day after preparation.

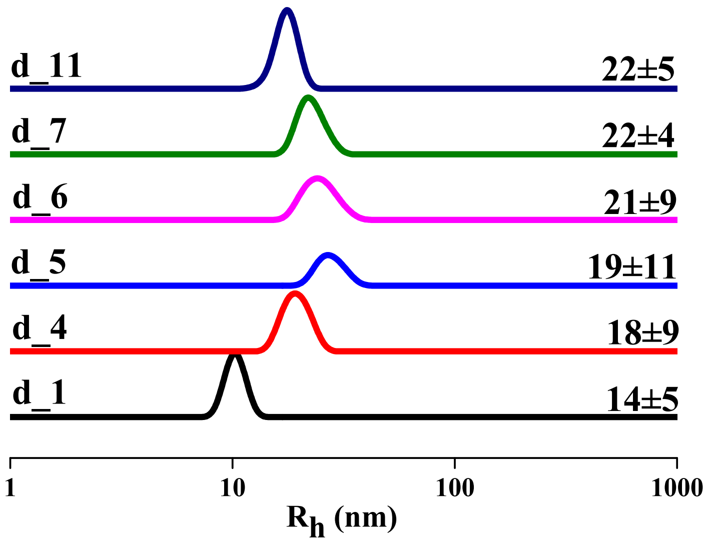

Figure S3. Hydrodynamic radius distribution over time of sample NPs:NaOl molar ratio 1:(2.1x10^6).

References

1. Luchini, A.; Gerelli, Y.; Fragneto, G.; Nylander, T.; Palsson, G. K.; Appavou, M.S.; Paduano, L. Neutron reflectometry reveals the interaction between functionalized SPIONs and the surface of lipid bilayers. *Colloids Surf. B* **2017**, *151*, 76-87.

2. Russo Krauss, I.; Picariello, A.; Vitiello, G.; De Santis, A.; Koutsioubas, A.; Houston, J. E.; Fragneto, G.; Paduano, L. Interaction with human serum proteins reveals biocompatibility of phosphocholine-functionalized SPIONs and formation of albumin-decorated nanoparticles. *Langmuir* **2020**, *36*, 8777-8791.
